# Outcomes of Participation in a Community-Based Physical Activity Program

**DOI:** 10.3389/fpubh.2019.00225

**Published:** 2019-08-14

**Authors:** Michelle Arnett, Sarah E. Toevs, Laura Bond, Elizabeth Hannah

**Affiliations:** ^1^Center for the Study of Aging, College of Health Sciences, Boise State University, Boise, ID, United States; ^2^Biomolecular Research Center, Boise State University, Boise, ID, United States; ^3^Independent Researcher, Boise, ID, United States

**Keywords:** older adult, rural, exercise/physical activity, social support, community-based services, health equity, public health partnership

## Abstract

Fit and Fall Proof^TM^ (FFP) program, established in 2004, is a peer volunteer-led collaboration between state and local public health organizations. The goal is to bring sustainable physical activity programs to underserved populations, including those in rural and frontier communities. FFP program is designed to help older adults maintain independence by improving mobility and function and providing opportunities for social engagement. The aim of this study was to evaluate the impact of participation in the program. A 6-month longitudinal study evaluated physical, social, and emotional outcomes among participants. The FES-I, SF-36v2, and Timed Up and Go (TUG) were collected. A convenience sample of new participants (*n* = 120, mean age = 75) representing rural and urban communities were recruited. FFP produced results similar to programs using physiotherapists or athletic trainers. Significant improvements were seen in TUG and SF-36v2 measures of physical, social, and emotional health. Participants completing at least one 10-week session (66%) demonstrated sustained improvements on these measures. While the average change in TUG between baseline and 10 weeks was statistically significant (*p* = 0.003), improvement in TUG was dependent on age and attendance. For participants <75 years, all attendance levels resulted in similar improvements in TUG. However, for those ≥75, improvements were strongly associated with the number of classes attended. Both the raw data and the model-based estimates of TUG times demonstrated that as age and attendance increase, greater improvements in TUG times were observed. The FFP program promotes health equity by reaching community-dwelling, underserved senior populations. The FFP program is in its 15th year and serves as an example of a sustainable collaboration between state and local public health organizations that is translatable to both urban and rural settings.

## Introduction

The population of older adults is growing at an unprecedented rate, outpacing the growth of the total US population (1). This demographic shift, along with the desire and need for older adults to age-in-place demands innovative partnerships between private and public health sectors. These collaborations are needed to bolster health promotion strategies designed to reduce loss of independence from falls, social isolation, and other modifiable risk factors.

The Centers for Disease Control and Prevention's (CDC) Healthy People 2020 recognizes fall prevention and increasing physical activity for older adults as public health priorities. Fall injuries are among the top 20 most expensive medical conditions, with costs to Medicare reaching $31 billion in 2015 (2). In Idaho, where this study was conducted, ~30% of Idahoans aged 65+ fall at least one time per year (3). Falling is a leading cause of unintentional injury and death among older adults in Idaho, with estimated fall-related medical costs reaching $253 million annually (4–6).

Indirect and quality of life costs of falling are high as well, including lost wages and travel expenses for fallers and their family caregivers. Quality of life costs include loss of independence, increased anxiety and fear of falling, social isolation, inability to perform daily tasks, and pain and suffering caused by falls (7–9).

## Identifying Fall Risks

There are numerous, interacting factors that influence a person's risk of falling, including age, medical conditions, and loss of balance and strength (8, 10, 11). Social isolation—living alone, small social network, and infrequent participation in social activities—is also associated with reduced physical activity and negative health consequences, including, depression, anxiety, and an increased likelihood of reporting fair or poor health (12–14).

The tracking of falls in a community-setting is difficult without access to medical records because self-reporting of falls is often underreported by older adults. One commonly used tool for assessing and identifying fall risks in clinical and community settings is the Timed Up-and-Go (TUG) (15, 16). It has been found to be a sensitive (sensitivity = 87%) and specific (specificity = 87%) tool for identifying those prone to falls.

## Fall Prevention Strategies

The CDC and National Council on Aging (NCOA) recognize a number of evidence-based programs designed to reduce falls while generating positive return on investment (ROI) (17, 18). Some interventions address multiple risk factors, such as Yale FICSIT and Stepping On (19, 20). Others, such as OTAGO Exercise Programme, are individually tailored exercise-based interventions lead by a physical or occupational therapist (21). And some, such as the A Matter of Balance program, are workshops using volunteer lay-leaders designed to reduce fear of falling and encourage physical activity levels among community-dwelling older adults (22).

A review of the literature revealed a “research-to-practice” gap with little information about the impact and sustainability of fall prevention programs outside the controls of a research model which allows for the direct tracking of falls (22–27). Li et al. (25) examined 14 exercise-based fall intervention programs identified in the CDC Compendium of Effective Fall Interventions and found only three to have information about implementation activities and only two providing details of training opportunities.

Two recent studies, a case study by Harnish et al. (28) and Li et al. (25), reported results of translational fall prevention interventions employing paid instructors in a senior living facility and senior center, respectively. Both studies demonstrated success in implementing research concepts into practice. However, for peer volunteer-led programs in community settings, a gap in the “research-to-practice” gap persists in frontier and rural areas.

## Fit and Fall Proof™ Program

Fit and Fall Proof™ (FFP), the focus of this study, is a theory-based, peer-led physical activity program for older adults designed to reduce risk of falling by improving function and mobility. The program is a collaboration between public health organizations and the private sector (community centers, churches, senior living centers, etc.). The goal is to bring sustainable physical activity programs to underserved populations, including those in rural and frontier communities. Established in 2004, FFP is based on principles of adult learning and the Social Cognitive Theory constructs of social support, normative beliefs (group physical activities); modeling, observational learning (peer leaders and classmates); and behavioral capacity and reinforcement (10-week class sessions, positive feedback from peers). A pre- and post-assessment of strength and mobility using the eight-foot Timed Up-and-Go (TUG) provides a personal measure of change (outcome expectation) as individuals develop behavioral capacity and increased self-efficacy (29).

The 10-week program delivered 2–3 times per week was developed and updated based on best practices to improve physical function and balance in older adults (30–34). The classes incorporate multiple categories of exercise (gait, balance and function; strength/resistance; and movements through three spatial planes) which have been shown to yield statistically significant reductions in fall rates (23). The program, detailed in the FFP Class Leader Training Manual, provides descriptions and pictures of key exercises and includes examples of how to modify exercises for a variety of functional skill levels (35). For example, classes are structured to include a warm-up using walking and range of motion exercises; a main set using gait and dynamic balance exercises and progressive resistance exercises using body weight and Therabands™; and a cool down of static balance and flexibility exercises.

The program is funded through a CDC block grant and state resources, and administered by FFP Coordinators located within Idaho's seven local health districts with oversight from the Idaho Department of Health and Welfare. It was designed specifically to reach the rural/frontier Idaho populations where access to exercise facilities, physiotherapists, or certified personal trainers is limited.

Core Master Trainers have doctoral or graduate degrees in kinesiology and physical education and extensive knowledge and experience in physical activity for older adults. Master Trainers must meet specific qualifications including demonstrated knowledge of anatomy and physiology, experience with providing instruction to older adults, and familiarity with the FFP program. The Master Trainers work closely with the local health district FFP coordinators to ensure consistency in instruction and program fidelity across the state. Health districts, through agreements with the state, are required to maintain appropriate class sites, purchase class materials and supplies, provide oversight of class leaders and Master Trainers. District FFP Coordinators and Master Trainers are required to attend an annual 1-day Refresher Workshop lead by the Core Master Trainers. Master Trainers teach the volunteer class leaders using the Manual as their textbook. Volunteers practice the exercises and lead segments of a FFP class during the training to develop their skills as a class leader.

Coordinators conduct quarterly site visits to ensure program fidelity and to manage process and outcome evaluation measures. Data collection at each site includes location, participant names, attendance records, and pre- and post- TUG scores. Class leaders collect TUG scores for all new participants and at least twice annually for ongoing participants. Records are reviewed by the coordinators, submitted to the state FFP coordinator, and analyzed quarterly and annually to monitor impact and guide program improvements.

## Research Aims

The objective of this study was to evaluate the impact of the Idaho FFP program among new participants. The primary measurement tools were the TUG test and the SF-36v2. Secondary tools included the Falls Efficacy Scale International (FES-I) and a brief, opened-ended survey collected from participants at the end of a 10-week session.

## Design and Methods

**Figure 1:**
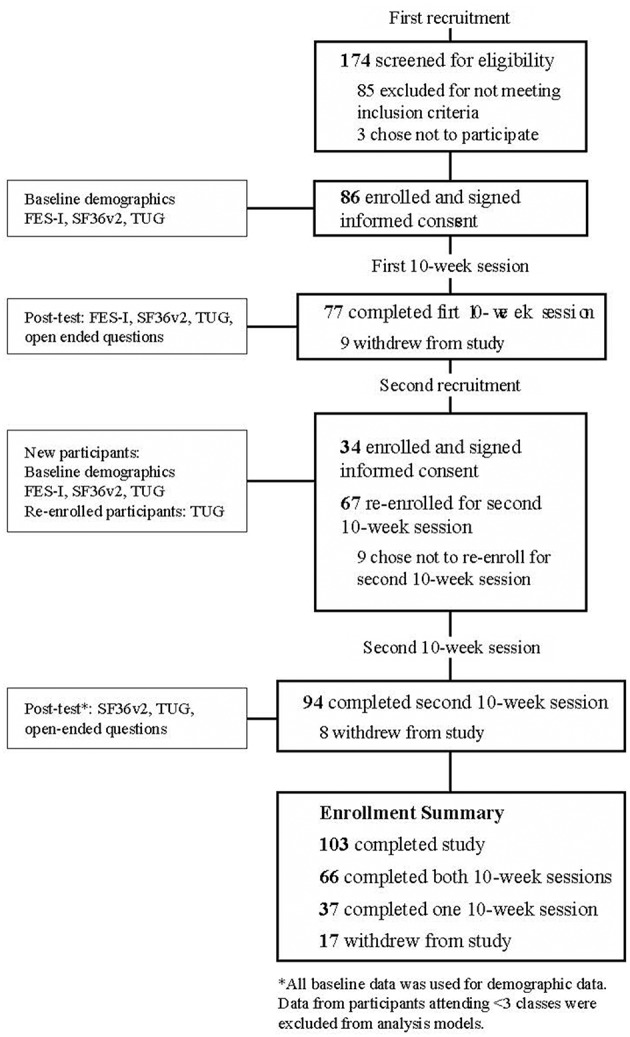
Enrollment flow and data collection sequence.

All research procedures were approved by the Boise State University Institutional Review Board. The Transparent Reporting of Evaluation with Non-randomized Designs (TREND) framework was used to guide study design and reporting (36); see Figure 1 for enrollment flow and data collection sequence.

To minimize disruption by the researchers, class leaders conducted the sessions without researcher involvement. Researchers obtained informed consent from interested individuals and distributed surveys in the first week and made follow-up visits at the completion of each of two 10-week sessions.

### Participants

Study participants in this longitudinal cohort study were identified from FFP classes in rural and urban settings in two waves and visited during the first week of the 10-week sessions conducted between September 3, 2014 and March 27, 2015. Individuals who were new to or returning after a >3-month hiatus from the program were recruited. No individual was excluded due to presence (or number) of chronic health conditions or because they used an assistive device for personal mobility.

Participants completed three baseline surveys; a demographic survey (e.g., age, gender, number of chronic disease conditions (disease burden), self-perceived health, use of assistive devices for walking), the Falls Efficacy Scale International [FES-I; (37)] and the SF-36v2 (38). Participants also provided a baseline eight-foot TUG. The SF-36v2 surveys and eight-foot TUG were re-administered at the end of each 10-week session. Additionally, a survey including open ended questions asking participants to share perceptions of FFP was administered at the end of the program.

### Measurements

The SF-36v2 instrument (38) uses 36 questions to establish the following eight domain scores: Physical Functioning (PF), Physical Role Limitations (RP), General Health (GH), Social Functioning (SF), Emotional Role Limitations (RE), Bodily Pain (BP), Mental Health (MH), and Vitality (VT). The survey also provides a Physical Composite Score (PCS) and Mental Composite Score (MCS) to facilitate interpretation of the general burden of illness (38). Norm-based scoring was applied to the participants' responses per scoring software (38). Higher scores on all dimensions reflect “better” emotional and physical health. Published estimates of reliability for the instrument are 0.96 (95% CI ± 3.9) and 0.93 (95% CI ± 5.3) for the PCS and MCS respectively, with the eight domains ranging from 0.82–0.96 (95% CI ± 3.9–8.3).

The eight-foot TUG test is a frequently used functional fitness assessment for community residing older adults. The TUG score represents the time (in seconds) it takes to stand up from a chair, walk eight-feet around a cone, and return to the chair. The test has a published test-retest reliability of 0.95 (16, 39, 40). Rikli and Jones (41) identified TUG scores that correlate with ability to remain mobile and functionally independent. Lower values indicate faster times to perform the activity and any improvement in time indicates greater mobility and reduced risk of falling (16).

### Data Analysis

For the 11 outcomes studied (TUG, MCS, PCS, and the eight domains), we validated that missing data did not create apparent bias by fitting a logistic regression on the presence of data as a function of the predictors to be used. To determine whether participants demonstrated improvements in the outcomes, we used a hierarchical linear model to account for repeated measures of participants, and correlation among participants within locations, using a compound symmetric structure for both variance components.

Using this framework for the 10 SF-36v2 outcomes, we regressed the outcome on test (baseline, first 10-week session, or second 10-week session), age, attendance (number of classes per session), and interactions for age by test, and age by attendance. This was the fullest model that allowed estimation of outcomes at all test times, was appropriate based on residual plots and Akaike's Information Criterion adjusted for small sample size (AIC_*C*_) (42), and addressed our specific research questions. All models were assessed for appropriateness using residual plots and identification of outlying and influential observations. For TUG, we used a similar model but based on residual plots and AIC_*C*_, we log-transformed the times. Age and attendance per session were mean-centered to facilitate estimation and model fitting. For presentation, the model-based log TUG estimates and standard errors were back-transformed to the original scale using methods based on Jørgensen and Pedersen (43).

These models were used to estimate and compare outcomes at the three testing times; for each outcome, we adjusted the comparisons using a Hochberg adjustment (44). We used our model for TUG to estimate and compare the 10-week TUG for different age and attendance levels. We did not use data from six participants who attended fewer than three classes in either 10-week session in any of these models (SF-36 outcomes and TUG).

We also considered whether gender, urban or rural setting, disease burden, or use of assistive devices would change results, especially whether these would alter the response over the course of the three assessments. For each outcome and each of these four variables, we added the variable as a main effect to the existing model. Additionally, we interacted the variable with assessment (baseline, first 10-weeks, second 10-weeks). Using AIC_*C*_, we addressed whether better models resulted, and whether the interaction was an added improvement. We considered models within two AIC_*C*_ units to be comparable, and highlighted results with AICC better than two less than the base model and when the overall *p*-value of the effect was <0.15 (45).

We compared the baseline responses of the 12 outcomes studied, plus disease burden and general health between those who did and did not complete a 10-week session using a t-test, and a chi-square test for use of assistive devices. No statistically significant differences were identified.

Statistical analysis was completed with IBM SPSS Statistics, Version 22.0 (46), SAS 9.4. (47), and R 3.5.0 for graphics (48). Raw SF-36v2 survey results were imported into the QualityMetric Health Outcomes^TM^ Scoring Software 4.5 to obtain age and gender-based results for the eight domains and the two composite scores. Statistical significance was set at *p* < 0.05.

The open-ended survey responses were entered into a Microsoft Excel (2013) database. We conducted frequency counts and used the Key Word In Context method to identify themes and develop codes (49, 50). An iterative process was used to assure that the coding was consistent with the data and revisions were made as necessary.

## Results

Participants (*n* = 120) were recruited from 10 sites in southwestern Idaho. Two of the sites were rural with 64 participants (53.0%) and the remaining eight were urban with 56 participants (47.0%). Of the 120 participants, 66 (55%) completed two 10-week sessions (20 weeks), and 37 (30.8%) completed one 10-week session. Seventeen (14.2%) withdrew from the study due to scheduling conflicts and lack of interest in continuing in the study. The baseline population included 95 women (79.2%) and 25 men (20.8%). There were 63 participants aged 75 (52.5%) and over. Of the 57 (47.9%) participants aged ≤74, over half (63.2%) were from rural locations. Table 1 describes baseline demographic and health status characteristics of participants, overall and by age group. While 102 (85%) self-reported baseline health as good or better, 47 (39%) had three or more chronic conditions and 17 (14.2%) reported no chronic conditions.

**Table 1:** Baseline demographic and health status characteristics overall and by age group.

	**All Study participants** **(*N* = 120)**	**Participants <75 years** **(*N* = 57)**	**Participants 75 + years** **(*N* = 63)**
Female Mean Age, *n =* 95	76.5 (54–93)	68.5 (54–74)	83.3 (76–93)
Male Mean Age, *n =* 25	74.7 (49–94)	67.6 (49–74)	82.4 (75–94)
Timed Up and Go mean score (range)	7.8 (4.0–21.9)	6.03 (4.19–9.13)	8.16 (3.87–25.50)
FES-I score (range)	23.3 (16.0–57.0)	22.6 (16.0–47.0)	24 (16.0–57.0)
Self-reported Health Status			
Excellent/Very Good/Good	84.9%	87.7%	82.3%
Fair or Poor	15.1%	12.3%	17.7%
Self-Reported Use of Assistive Device			
None	84.2%	93.0%	77.8%
Cane	10.8%	5.3%	5.3%
Walker	5.0%	1.8%	1.8%
Self-Reported chronic conditions			
Arthritis	52.5%	47.4%	57.1%
Hypertension	44.3%	33.3%	54.0%
Osteoporosis	17.5%	15.8%	19.0%
Heart Disease	16.7%	5.3%	27.0%
Diabetes	15.0%	15.8%	14.3%
Lung disease	13.3%	19.3%	7.9%
Cancer	13.3%	8.8%	17.5%
Depression/Anxiety	11.7%	15.8%	7.9%
Stroke	5.0%	3.5%	6.3%
Disease Burden: number of reported chronic conditions			
None	14.2%	21.1%	7.9%
1–2	46.7%	45.6%	47.6%
3–4	36.7%	31.6%	41.3%
5 or more	2.5%	1.8%	3.2%

*Those over 75 years have a slightly higher number of chronic conditions (p = 0.056, Cochran-Mantel-Haenszel test)*.

There was no difference at baseline between those who did and did not complete a 10-week session by age, self-perceived health, TUG, FES-I, use of an assistive device, disease burden, or SF-36v2 results.

The range of FES-I Score responses at baseline was 16–57 points with no difference between scores for males and females (*p* = 0.39). The baseline average FES-I score for participants was 23.3 (±7.1) indicating a moderate concern about falling based on cut-points identified by Delbaere et al. (37).

For the first 10-week session, average attendance was 12.9 (±5.7) classes, or 62.5% of available classes. For the second 10-week session, average attendance was 15.5 (±5.7), or 64.5% of the available classes.

From the modeling results, outcomes with statistically significant test effects were TUG, PF, VT, RE, SF, MH, and MCS (Table 2). Age was statistically significant for TUG, PF, MH, and MCS, and the age by test interaction was significant for TUG. Attendance was not statistically significant as a main effect for any outcome but interacted with age for TUG. The SF-36 outcomes RP, GH, BP, and PCS had no statistically significant terms in the models.

**Table 2:** Model-adjusted estimated means^a^ and standard errors from the hierarchical model at baseline, 10 weeks, and 20 weeks.

				***p*****-values**^**b**^**, Baseline to:**	
**Outcome**	**Baseline**	**10-week**	**20-week**	**1st 10-weeks**	**2nd 10-weeks**
TUG^c^	6.7 (±0.3)	6.2 (±0.3)	6.1 (±0.3)	0.000	0.000
PF	42.2 (±0.8)	45.4 (±0.8)	45.0 (±0.9)	0.000	0.002
RP	44.2 (±1.2)	45.2 (±1.3)	44.7 (±1.4)	0.656	0.656
GH	52.3 (±0.7)	52.9 (±0.7)	52.6 (±0.8)	0.761	0.761
SF	49.1 (±1.1)	51.6 (±1.1)	50.7 (±1.2)	0.007	0.179
RE	46.8 (±1.3)	49.0 (±1.4)	50.1 (±1.5)	0.061	0.013
BP	47.6 (±0.9)	48.3 (±0.9)	49.3 (±1.0)	0.445	0.232
MH	51.8 (±1.1)	53.8 (±1.2)	53.9 (±1.2)	0.007	0.014
VT	51.5 (±1.1)	53.3 (±1.1)	52.6 (±1.2)	0.022	0.281
PCS	44.6 (±0.8)	46.1 (±0.8)	45.8 (±0.9)	0.085	0.267
MCS	52.4 (±1.2)	54.4 (±1.2)	54.5 (±1.3)	0.018	0.027
FES	24.4 (±1.2)	24.2 (±1.2)	25.2 (±1.2)	0.705	0.277

a Model-adjusted means for each test period, calculated at the mean age and mean attendance for each period.

b Comparisons between baseline and each test period, and between test periods, were adjusted separately for each outcome using Hochberg adjustment (44).

c *Estimates for participants without assistive devices; use of these devices adds 0.46 (±0.06) seconds to all testing times. Model-adjusted means for each test period, calculated at the mean age and mean attendance for each period. Means compared between test periods, within each outcome, and adjusted for multiple comparisons using Hochberg adjustment. TUG, Timed-Up and Go; PF, Physical Functioning; RP, Physical Role Limitations; GH, General Health; SF, Social Functioning; ER, Emotional Role Limitations; BP, Bodily Pain; MH, Mental Health; VT, Vitality; PCS, Physical Composite Score; MCS, Mental Composite Score; FES, Falls Efficacy Scale International*.

Model-based estimates and changes in outcomes from baseline to the first and second 10-week sessions, calculated at the average age (76 years) and attendance (first session 12.9 classes, second session 15.5 classes) are reported in Table 3. Overall, the mean TUG time at baseline was 6.7 seconds (SD 0.3). For those participating in a 10-week session a statistically significant decrease in TUG time was observed, to 6.2 seconds (SD 0.3). For those enrolling in an additional 10-week session the estimated time was 6.1 (SD 0.3) s. Of the 10 SF-36v2 outcomes, most improved over the course of the first 10-week session. Four of the five mental health domains showed statistically significant improvements in either the first or the second 10 week periods with the composite score showing improvement in both. None of the outcomes displayed a change between the first 10-week testing period and the second indicating sustained gains.

**Table 3:** Model-based TUG times, seconds (95% confidence interval).

		**10-week TUG**			**Difference from baseline, seconds**		
**Age**	**Baseline**	**10 classes**	**13 classes**	**21 classes**	**10 classes**	**13 classes**	**21 classes**
67 years	6.4 (5.6, 7.2)	5.6 (4.9, 6.3)	5.6 (5.0, 6.4)	5.8 (5.0, 6.6)	0.8	0.8	0.6
77 years	7.3 (6.6, 8.1)	6.8 (6.2, 7.6)	6.8 (6.1, 7.5)	6.6 (5.9, 7.3)	0.5	0.5	0.7
86 years	8.2 (7.3, 9.2)	8.1 (7.2, 9.1)	7.9 (7.0, 8.9)	7.3 (6.5, 8.3)	0.1	0.3	0.9

*Model-based times show that the benefit of attendance is proportionally greater for older participants*.

After controlling for the test, age, and attendance, each additional chronic condition significantly decreased SF-36 scores for all physical health outcomes (PF, RP, GH, BP, and PCS). Chronic conditions also decreased scores in the mental health outcomes SF and RE. Those using assistive devices had lower scores on all physical health domains and on the social health domains SF and VT (see Supplemental Data). The physical health outcome BP initially scored lower on the baseline assessment, but the scores for this domain increased for each following assessment. Scores on the composite PCS were quite low on the baseline and second 10-week assessment, but rose for the first 10-week assessment (Table 4).

**Table 4:** Change in outcomes for each additional chronic condition (Disease Burden) and use of assistive devices.

**Outcome**	**Disease burden model-based estimate (SE)**	**Assistive devices model-based estimate (SE)**
PF	−1.5 (0.6)	−11 (1.9)
RP	−1.8 (0.5)	−8.7 (1.9)
BP	−2.2 (0.6)	−8.0 (2.4),−1.5 (2.6),−4.7 (2.9)^*^
GH	−2.0 (0.5)	−6.7 (2.0)
PCS	−2.2 (0.5)	−13 (2.1),−7.2 (2.3),−11 (2.5)^*^
VT		−5.0 (2.0)
SF	−1.2 (0.5)	−7.6 (1.7)
RE	−1.1 (0.5)	
MH		
MCS		
Log(TUG)		0.4 (0.1)

* *Three estimates indicate the interaction was significant. Estimates for each assessment period (baseline, first 10-weeks, second 10-weeks) reported*.

MH also had a statistically significant decrease with each added chronic condition, but this drop was nearly erased by the second 10-week assessment. Those using devices had lower scores on all physical health domains, and for two of those (BP and PCS), the scores declined with each additional test. This was also true for the social health domains SF and VT. Women scored significantly higher than men on the SF domain. Rural residents scored statistically higher than urban participants on the social health domains MCS, MH, SF, and VT, and on the physical health domain RP, on at least one of the assessments (baseline, first 10 weeks, or second 10 weeks).

**Figure 2:**
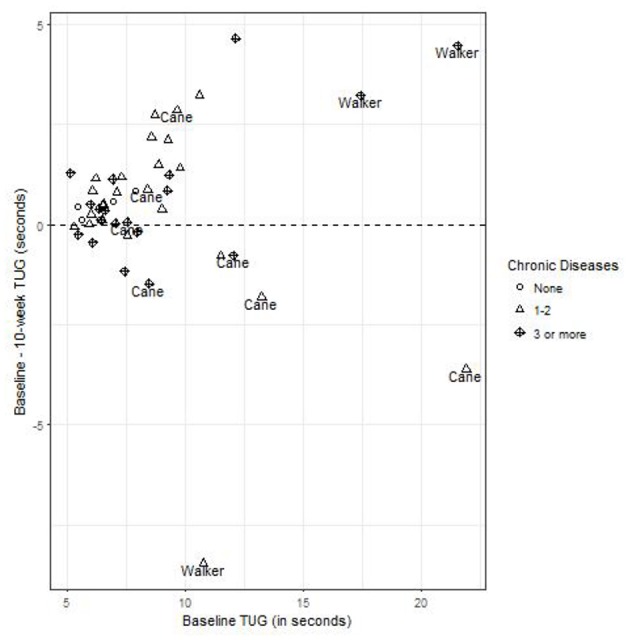
The change in 10-week TUG times, as a function of baseline TUG, identifying chronic conditions, and assistive devices. Points above the horizontal line at 0 have a faster 10-week TUG than baseline, and the distribution of points around this line are equivalent for those with and without chronic conditions, and with or without assistive devices.

There was no association between number of chronic diseases and TUG baseline time, and change over the testing period (Figure 2). TUG times were significantly slower for individuals with assistive devices but this did not statistically change with subsequent assessments. TUG times were not altered by the disease burden of participants.

**Figure 3:**
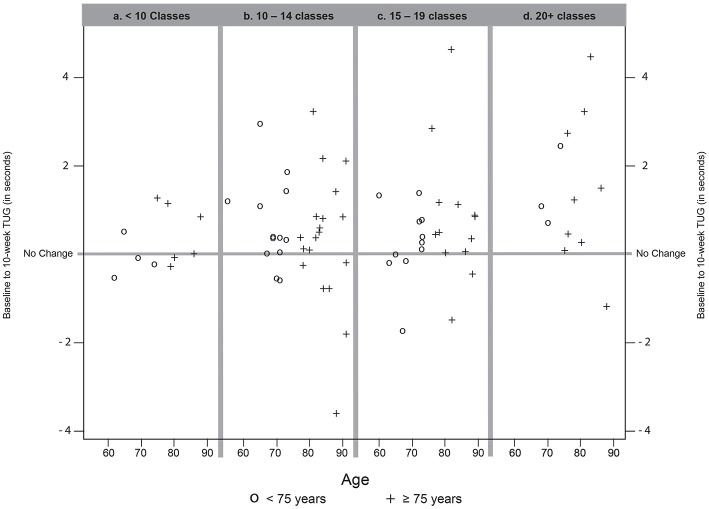
Change from baseline to 10-week TUG, by (a) participants attending fewer than 10 Classes, (b) participants attending 10–14 classes, (c) participants attending 15–19 classes, and (d) participants attending 20 or more classes during the 10 week session. Positive results (above the No Change line) indicate a faster individual TUG at follow-up compared to baseline. Negative results (below the No Change line) indicate a slower individual TUG at follow-up compared to baseline. One participant (over 75 years, attendance <10 Classes) whose TUG increased by 8 seconds is not shown to facilitate the plotting scale. Symbols identify participants age group as <75 years (O) or 75 years and older (+).

While the average change in TUG between baseline and 10 weeks was statistically significant (*p* = 0.003), improvement in TUG was dependent on age and attendance. For participants <75 years, all attendance levels resulted in similar improvements in TUG. However, for those ≥75, improvements were strongly associated with the number of classes attended. Both the raw data (Figure 3) and the model-based estimates of TUG times displayed in Table 3 show that as age and attendance increase, the model identified proportionately greater improvements in TUG times.

Seventy-two participants answered the open-ended questions reporting their perceptions of the classes. While participants enjoyed the exercises and instructors, the most common self-reported benefit (37% of responses) was the opportunity for social engagement as evidenced by the following comments:

“Getting to be around positive people.”

“Visiting with friends while getting to exercise.”

“The people, laughter and friendships we share.”

“I look forward to seeing everyone each week and socializing.”

Some participants provided suggestion for program improvement, with the most common being a request that instructors add more variety in exercise routines (28%) and for more classes each week (12%).

## Discussion and Implications

The aim of this study was to determine whether the FFP program achieves its goals of increasing mobility, function, and social engagement for participants. Based on our primary outcomes, FFP demonstrated results consistent with other community-based fall prevention programs using physiotherapists and other certified, and often paid, trainers (22, 27, 51). Findings from this study are also consistent with a systematic review and meta-analysis by Burton et al. (52) demonstrating improvements in function based on TUG and other functional tests.

Findings indicate that the FFP program provides functional benefits, based on improved TUG times, as well as improved Physical Functioning (PF) and PCS scores. The MCS, MH, VT, and SF also demonstrated significant improvements in social/emotional health over time when controlling for assistive devices or chronic conditions. Although norms for TUG have been established (40, 53), we did not compare our population to these because the parameters are based on populations that did not use assistive devices. Nor did we use SF36v2 norms because of inconsistencies across domains with our study population.

Evidence that TUG improvements gained over the first 10-week session were sustained during the second session reinforces that these changes can be attributed to participation in the FFP program. The benefits of participating in the FFP program were particularly evident among those who were older, reported a higher number of chronic conditions, or slower on their baseline TUG (Figure 2). Program benefits were also shown to be related to the frequency of attendance, particularly among participants over the age of 75 (Table 3).

Individuals with chronic disease experience significantly higher rates of falling than those without a chronic condition (54, 55). Although chronic diseases are not normally considered as a modifiable risk factor, we found that participants with multiple chronic conditions remained engaged in the program and made significant improvements in physical, social, and emotional health.

## Limitations

The results of this study must be interpreted with caution due to the lack of a control or comparison group. This limitation is mitigated somewhat by the use of a longitudinal cohort study design which allowed us to draw inferences by monitoring change over time and demonstrating baseline equivalencies (56).

The use of the SF-36v2 survey in this population may also be a limitation due to a lack of consistency noted across domains for individuals. For example, an individual who said that they had “A lot” of limitations climbing stairs also selected “No limitations” in vigorous activities such as running. We also found that some SF-36v2 questions were not applicable to our population. e.g., RP (role of physical health limitations), asks about health affecting employment, which did not apply to most of our participants.

In addition, the community-based nature of the intervention, budget, and privacy considerations limited our ability to collect information on falls among FFP participants. Based on a recent review, we can assume a reduction in fall risk because FFP includes functional exercises and balance training at each class (57).

Current studies of fall prevention/physical activity programs and validated tools are developed for use with generally healthy older adults. Future research is needed to establish the effectiveness of these programs involving diverse populations, i.e., those with limited mobility (canes, wheelchairs, etc.) and high disease burden.

## Implications

There are numerous studies examining the effects of fall prevention strategies. But there are few evaluations of long-term, community-based programs using the train-the-trainer model with older adults as class leaders. This study provides triangulated evidence that participants, regardless of age, gender, disease burden, or physical limitations receive significant physical and psychological benefits from participation in the FFP program. The 15-year history of the delivery of FFP addresses the research-to-practice gaps identified in the literature (25, 26). Specifically, it provides an implementation plan that includes an evidenced-based curriculum and outlines process and outcome evaluation strategies.

This program also aligns with the recommendations from a meta-analysis by Sherrington et al. (57) that physical activity programs should be conducted at least 2 h per week on an ongoing basis. Interventions should challenge balance, improve function and target the general community, including those at high risk for falling (chronic conditions, assistive devices, etc.).

The FFP program promotes health equity by reaching underserved populations. Older Idahoans who are low-income or reside in rural areas often lack access to gyms, athletic trainers, and fee-based exercise programs. Bridging this gap provides needed social interaction and exercise opportunities for many seniors. By actively engaging in community partnerships with senior centers, churches, and other organizations that offer facilities for FFP classes, and by mobilizing and training volunteer peer instructors to lead the classes, FFP provides a supervised, evidence-based curriculum at no cost to participants.

Participation in the program promotes healthy aging, allowing many Idahoans to age in place and avoid or delay institutionalization, regardless of income or location. This partnership between the private and public health sectors demonstrates the ability to bring sustainable, critical physical activity programming to rural and frontier communities.

## Data Availability

The raw data supporting the conclusions of this manuscript will be made available by the authors, without undue reservation, to any qualified researcher.

## Ethics Statement

This study was carried out in accordance with the recommendations of the U.S. Department of Health and Human Services (HHS) Office for Human Research Protections with written informed consent from all subjects. All subjects gave written informed consent in accordance with the Declaration of Helsinki. The protocol was approved by the Boise State University Institutional Review Board.

## Author Contributions

MA: lead author, study conception and design, manuscript writing, and data collection and final approval of version being published. ST: study conception and design, manuscript writing, and final approval of version being published. LB: data analysis, manuscript writing, and final approval of version being published. EH: study conception and design.

### Conflict of Interest Statement

The authors declare that the research was conducted in the absence of any commercial or financial relationships that could be construed as a potential conflict of interest.

## References

[1] Ortman JM Velkoff VA Hogan H An Aging Nation: The Older Population in the United States. Report Number: P25-1140. Washinton, DC: United States Census Bureau (2014).

[2] Burns ER Stevens JA Lee R. The direct costs of fatal and non-fatal falls among older adults - United States. J Saf Res. (2016) 58:99–103. 10.1016/j.jsr.2016.05.001 27620939 PMC6823838

[3] Bergen G Stevens M Burns E. Falls and fall injuries among adults aged ≥65 years - United States, 2014. MMWR Morb Mortal Wkly Rep. (2016) 65:993–8. 10.15585/mmwr.mm6537a2 27656914

[4] CDC Cost of Falls Among Older Adults. (2016). Retrieved from: http://www.cdc.gov/homeandrecreationalsafety/falls/fallcost.html (accessed November 16, 2019).

[5] Idaho Department of Health and Welfare Facts About Falls (n.d.). Retrieved from: http://healthandwelfare.idaho.gov/Health/IdahoPhysicalActivityandNutrition(IPAN)/FitandFallProof%E2%84%A2/tabid/199/Default.aspx (accessed November 1, 2019).

[6] Mirel LB Carper K Trends in health care expenditures for the elderly, age 65 and over: 2001, 2006, and 2011. In: Statistical Brief (Medical Expenditure Panel Survey (US)). Rockville, MD: Agency for Healthcare Research and Quality (2014). Available online at: http://www.meps.ahrq.gov/mepsweb/data_files/publications/st429/stat429.shtml.

[7] Blumenthal JA Babyak MA Moore KA Craighead WE Herman S Khatri P. Effects of exercise training on older patients with major depression. Arch Internal Med. (1999) 159:2349–56. 10.1001/archinte.159.19.2349 10547175

[8] Miller RR Berry SD. Falls: epidemiology, pathophysiology, and relationship to fracture. Curr Osteoporosis Rep Curr Osteoporosis Rep. (2008) 6:149–54. 10.1007/s11914-008-0026-4 19032925 PMC2793090

[9] Stubbs B Stubbs J Gnanaraj SD Soundy A. Falls in older adults with major depressive disorder (MDD): a systematic review and exploratory meta-analysis of prospective studies. Int Psychogeriatr IPA. (2016) 28:23–30. 10.1017/S104161021500126X 26234532

[10] Ponce M How to prevent falls among older adults in outpatient settings. Am Nurse Today. (2012) 7.

[11] Rubenstein LZ. Falls in older people: epidemiology, risk factors and strategies for prevention. Age Ageing. (2006) 35:ii37–41. 10.1093/ageing/afl084 16926202

[12] Cornwell EY Waite LJ. Social disconnectedness, perceived isolation, and health among older adults. J Health Soc Behav. (2009) 50:31–48. 10.1177/002214650905000103 19413133 PMC2756979

[13] Liffe S Kharicha K Harari D Swift C Gillmann G Stuck AE Health risk appraisal in older people 2: the implications for clinicians and commissioners of social isolation risk in older people. Br J Gen Pract. (2007) 57:277–82. 17394730 PMC2043334

[14] Nicholson NR Jr. Social isolation in older adults: an evolutionary analysis. J Adv Nurs. (2009) 65:1342–52. 10.1111/j.1365-2648.2008.04959.x 19291185

[15] CDC Assessment: Timed Up and Go. (2017). Retrieved from: https://www.cdc.gov/steadi/pdf/STEADI-Assessment-TUG-508.pdf (accessed November 16, 2019).

[16] Shumway-Cook A Brauer S. Predicting the probability for falls in community-dwelling older adults using the Timed Up and Go Test. Phys Ther. (2000) 80:896–903. 10960937

[17] NCOA Learn About Evidence-Based Falls Prevention Programs (n.d.). Retrieved from: https://www.ncoa.org/healthy-aging/falls-prevention/falls-prevention-programs-for-older-adults/ (accessed November 16, 2019).

[18] Stevens JA Burns E A CDC Compendium of Effective Fall Interventions: Preventing Falls: What Works for Community-Dwelling Older Adults. 3rd ed (2015). Retrieved from: http://www.cdc.gov/homeandrecreationalsafety/Falls/compendium.html (accessed October 18, 2019)

[19] Clemson L Kendig H Mackenzie L Browning C. Predictors of injurious falls and fear of falling differ: an 11-year longitudinal study of incident events in older people. J Aging Health. (2015) 27:239–56. 10.1177/0898264314546716 25117181

[20] Tinetti ME Baker DI McAvey G Claus EB Garrett P. A multifactorial intervention to reduce the risk of falling among elderly people living in the community. N Engl J Med. (1994) 331:821–7. 10.1056/NEJM199409293311301 8078528

[21] Robertson MC Devlin N Gardner MM Campbell AJ. Effectiveness and economic evaluation of a nurse delivered home exercise programme to prevent falls 1: Randomised controlled trial. Br Med J. (2001) 322:697–701. 10.1136/bmj.322.7288.697 11264206 PMC30094

[22] Healy TC Peng C Haynes MS McMahon EM Botler JL Gross L The feasibility and effectiveness of translating a matter of balance into a volunteer lay leader mode. J Appl Gerontol. (2008) 27:18 10.1177/0733464807308620

[23] Gillespie LD Robertson MC Gillespie WJ Sherrington C Gates S Clemson LM Interventions for preventing falls in older people living in the community. Cochrane Database Syst Rev. (2012) CD007146 10.1002/14651858.CD007146.pub3 PMC8095069 22972103

[24] Layne JE Sampson SE Mallio CJ Hibberd PL Griffith JL Das SK. Successful dissemination of a community-based strength training program for older adults by peer and professional leaders: the people exercising program. J Am Geriatri Soc. (2008) 56:2323–9. 10.1111/j.1532-5415.2008.02010.x 19112654

[25] Li F Eckstrom E Harmer P Fitzgerald K Voit J Cameron KA. Exercise and fall prevention: narrowing the research-to-practice gap and enhancing integration of clinical and community practice. J Am Geriatr Soc. (2016) 64:425–31. 10.1111/jgs.13925 26825429 PMC4760892

[26] Mallonee S Fowler C Istre GR. Bridging the gap between research and practice: a continuing challenge. Inj Prev. (2006) 12:357–9. 10.1136/ip.2006.014159 17170181 PMC2564411

[27] Waters DL Hale LA Robertson L Hale BA Herbison P. Evaluation of a peer-led falls prevention program for older adults. Arch Phys Med Rehabl. (2011) 92:1581–6. 10.1016/j.apmr.2011.05.014 21963125

[28] Harnish A Dieter W Crawford A Shubert TE. Effects of evidence-based fall reduction programing on the functional wellness of older adults in a senior living community: a clinical case study. Front Public Health. (2016) 4:262. 10.3389/fpubh.2016.00262 28066752 PMC5177607

[29] Bandura A Social Foundations of Thought and Action: A Social Cognitive Theory Upper. Saddle River, NJ: Prentice Hall (1986).

[30] Clark MA Lucett SC Sutton BC, editors. Chapter 16: Chronic health conditions and physical or functional limitations. In: NASM Essentials of Personal Fitness Training, 4 ed Baltimore, MD: Lippincott Williams and Wilkins, a Wolters Kluwer Business (2012). p. 393–430.

[31] Durstine JL American College of Sports Medicine ACSM's Exercise Management for Persons With Chronic Diseases and Disabilities. Champaign, IL: Human Kinetics (2009).

[32] Fiatarone Singh MA Exercise comes of age: rationale and recommendations for a geriatric exercise prescription. J Gerontol. Seri A. (2002) 57A:M262–82. 10.1093/gerona/57.5.M262 11983720

[33] Lorig KR Sobel DS Stewart AL Brown BW Bandura A Ritter P. Evidence suggesting that a chronic disease self-management program can improve health status while reducing hospitalization: a randomized trial. Med Care. (1999) 37:5–14. 10.1097/00005650-199901000-00003 10413387

[34] Nelson ME Rejeski WJ Blair SN Duncan PW Judge JO King AC. Physical activity and public health in older adults: recommendation from the American College of Sports Medicine and the American Heart Association. Med Sci Sports Exerc. (2007) 39:1435–45. 10.1249/mss.0b013e3180616aa2 17762378

[35] Mittleider J Gibson T Arnett M Fit and Fall ProofTM Class Leader Training Manual. 2nd ed Boise, ID: Idaho Department of Health and Welfare, Division of Public Health (2017).

[36] Des Jarlais DC Lyles C Crepaz N TREND Group. Improving the reporting quality of nonrandomized evaluations of behavioral and public health interventions: the TREND statement. Am J Public Health. (2004) 94:361–6. 10.2105/AJPH.94.3.361 14998794 PMC1448256

[37] Delbaere K Close JCT Mikolaizak AS Sachdev PS Brodaty H Lord SR. The Falls Efficacy Scale International (FES-I). A comprehensive longitudinal validation study. Age Aging. (2010) 39:210–6. 10.1093/ageing/afp225 20061508

[38] Saris-Baglama RN Dewey CJ Chosholm GB Plumb E King J Rasicot P QualityMetric Health Outcomes™ Scoring Software 4.5 User's Guide. Lincoln, RI: Quality Metric Incorporated (2011).

[39] Podsiadlo D Richardson S The timed Up and Go: a test of basic functional mobility for frail elderly persons. J Am Geriatr Soc. (1991) 39:142–8. 10.1111/j.1532-5415.1991.tb01616.x 1991946

[40] Rikli RE Jones CJ Development and validation of a functional fitness test for community-residing older adults. J Aging Phys Activity. (1999) 7:129–61. 10.1123/japa.7.2.129

[41] Rikli RE Jones CJ. Development and validation of criterion-referenced clinically relevant fitness standards for maintaining physical independence in later years. Gerontologist. (2013) 53:255–67. 10.1093/geront/gns071 22613940

[42] Burnham KP Anderson DR Model Selection and Multimodel Inference: A Practical Information-Theoretic Approach. 2nd ed New York, NY: Springer (2002).

[43] Jørgensen E Pedersen AR How to Obtain Those Nasty Standard Errors From Transformed Data - and Why They Should Not be Used. Aarhus: Aarhus University (1998).

[44] Hochberg Y A sharper Bonferroni procedure for multiple significance testing. Biometrika. (1988) 75:800–2. 10.2307/2336325

[45] Arnold TW Uninformative parameters and model selection using Akaike's Information Criterion. J Wildlife Manag. (2010) 76:1175–8. 10.1111/j.1937-2817.2010.tb01236.x

[46] IBM Corp IBM SPSS Statistics for Windows, Version 24.0. Armonk, NY: IBM Corp (2013).

[47] SAS Institute Inc SAS/ACCESS^®^ 9.4 Interface to ADABAS: Reference. Cary, NC: SAS Institute, Inc (2013).

[48] Wickham H ggplot2: Elegant Graphics for Data Analysis. 2nd ed New York, NY: Springer International Publishing (2016). 10.1007/978-3-319-24277-4

[49] Elo S Kyngas H. The qualitative content analysis process. J Adv Nurs. (2008) 62:107–15. 10.1111/j.1365-2648.2007.04569.x 18352969

[50] Vaismoradi M Turunen H Bondas T. Content analysis and thematic analysis: Implications for conducting a qualitative descriptive study. Nurs Health Sci. (2013) 15:398–405. 10.1111/nhs.12048 23480423

[51] Ory MG Smith ML Jiang L Lee R Chen S Wilson AD. Fall prevention in community settings: results from implementing stepping on in three states. Front Public Health. (2015) 2:232. 10.3389/fpubh.2014.00232 25964924 PMC4410346

[52] Burton E Farrier K Hill KD Codde J Airey P Hill AM. Effectiveness of peers in delivering programs or motivating older people to increase their participation in physical activity: systematic review and meta-analysis. J Sports Sci. (2018) 36:666–78. 10.1080/02640414.2017.1329549 28535358

[53] Rikli RE Jones CJ Functional fitness normative scores for community-residing adults ages 60-94. J Aging Phys Activity. (1999) 7:162–181. 10.1123/japa.7.2.162

[54] Lee PG Cigolle C Blaum C. The co-occurrence of chronic diseases and geriatric syndromes: the health and retirement study. J Am Geriatri Soc. (2009) 57:511–6. 10.1111/j.1532-5415.2008.02150.x 19187416

[55] Voth J Straus S Munce S Sibley K Jaglal S. Chronic disease and falls in community-dwelling Canadians over 65 years old: a population-based study exploring associations with number and pattern of chronic conditions. BMC Geriatr. (2014) 14:1–11. 10.1186/1471-2318-14-22 24529293 PMC3928582

[56] Shadish WR Cook TD Campbell DT Experimental and Quasi-Experimental Designs for Generalized Causal Inference. Belmont, CA: Wadsworth/Cengage Learning (2002).

[57] Sherrington C Fairhall NJ Tiedemann A Michaleff ZA Howard K Clemson L. Exercise for preventing falls in older people living in the community. Cochrane Database Syst Rev. (2019) CD012424. 10.1002/14651858.CD012424.pub2 30703272 PMC6360922

