# Supplementary figures and images for: Outcomes of Participation in a Community-Based Physical Activity Program
Source: Front Public Health. 2019 Aug 14;7:225. doi: 10.3389/fpubh.2019.00225 (PMC6702613; doi:10.3389/fpubh.2019.00225)

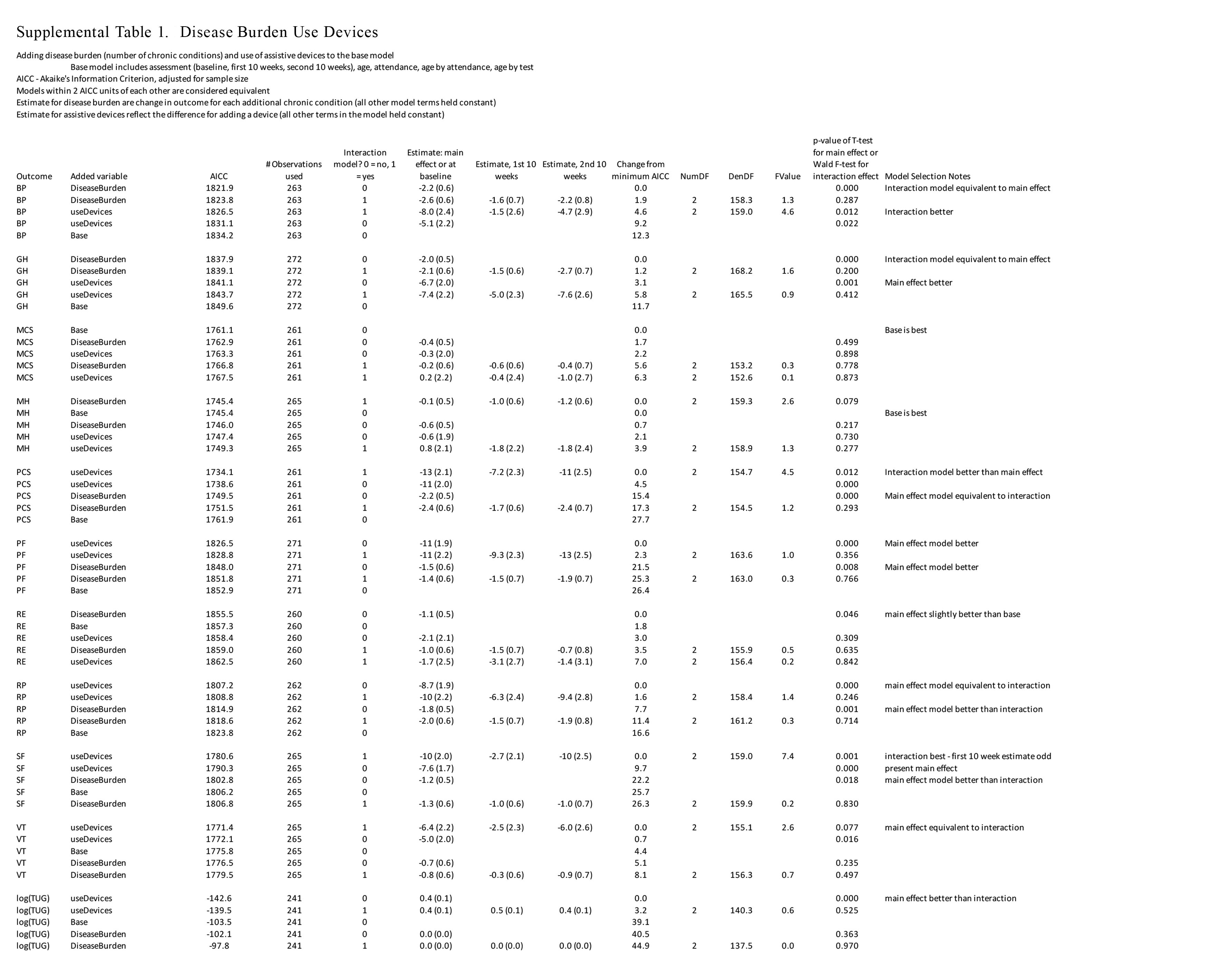

Supplement: Supplementary file 1 [file Image_1.JPEG]
